# The Assessment of Antimicrobial and Anti-Biofilm Activity of Essential Oils against *Staphylococcus aureus* Strains

**DOI:** 10.3390/antibiotics12020384

**Published:** 2023-02-13

**Authors:** Caglar Ersanli, Athina Tzora, Ioannis Skoufos, Konstantina Fotou, Eleni Maloupa, Katerina Grigoriadou, Chrysoula (Chrysa) Voidarou, Dimitrios I. Zeugolis

**Affiliations:** 1 Laboratory of Animal Science, Nutrition and Biotechnology, School of Agriculture, University of Ioannina, 47100 Arta, Greece; 2 Laboratory of Animal Health, Food Hygiene, and Quality, School of Agriculture, University of Ioannina, 47100 Arta, Greece; 3 Regenerative, Modular & Developmental Engineering Laboratory (REMODEL), Charles Institute of Dermatology, Conway Institute of Biomolecular and Biomedical Research and School of Mechanical and Materials Engineering, University College Dublin, D04 V1W8 Dublin, Ireland; 4 Laboratory of Conservation and Evaluation of Native and Floricultural Species, Institute of Plant Breeding; and Genetic Resources, Hellenic Agricultural Organization Demeter, Thermi, 57001 Thessaloniki, Greece

**Keywords:** antimicrobial resistance, essential oil, *Thymus sibthorpii*, *Origanum vulgare*, *Salvia fruticosa*, *Crithmum maritimum*, antimicrobial activity, anti-biofilm activity

## Abstract

The increase in antimicrobial resistance and tolerance over the years has become a serious public health problem, leading to the inevitable development of alternative antimicrobial agents as substitutes for industrial pharmaceutical antibiotics targeting humans and animals under the concept of one health. Essential oils (EOs) extracted from aromatic and pharmaceutical plants incorporate several bioactive compounds (phytochemicals) that positively affect human and animal health. Herein, this work aimed to examine a standardized chemical composition and screen the antimicrobial and anti-biofilm activity of *Thymus sibthorpii*, *Origanum vulgare*, *Salvia fruticosa*, and *Crithmum maritimum* EOs against three different *Staphylococcus aureus* strains by gold-standard disc diffusion, broth microdilution, and microtiter plate biofilm assays. Therefore, the evaluation of the above-mentioned EOs were considered as substitutes for antibiotics to combat the ever-mounting antimicrobial resistance problem. The observed bacterial growth inhibition varied significantly depending on the type and concentration of the antimicrobials. *Thymus sibthorpii* was determined as the strongest antimicrobial, with 0.091 mg/mL minimum inhibitory concentration (MIC) and a 14–33 mm diameter inhibition zone at 5% (*v*/*v*) concentration. All tested EOs indicated almost 95% inhibition of biofilm formation at their half MIC, while gentamicin sulfate did not show sufficient anti-biofilm activity. None of the methicillin-resistant strains showed resistance to the EOs compared to methicillin-sensitive strains. *Thymus sibthorpii* and *Origanum vulgare* could be potential alternatives as antimicrobial agents to overcome the problem of microbial resistance. The tested EOs might be incorporated into antimicrobial products as safe and potent antimicrobial and anti-biofilm agents.

## 1. Introduction

Increased consumption and misuse of antimicrobial agents in both humans and animals [1,2] have caused the spread of antimicrobial resistance, which seriously threatens public and animal health [3]. Whereas infections due to antimicrobial resistance exhibited by bacteria can be adaptive, intrinsic, and acquired [4], multidrug-resistant bacteria (e.g., methicillin-resistant *Staphylococcus aureus*) cause infections that end up with longer hospitalization periods, remarkable morbidity, and mortality [3,5], as well as high healthcare costs. According to a report from the Organization of Economic Cooperation and Development (OECD), approximately 2.4 million people are expected to die due to this kind of infection in North America, Australia, and Europe over the next three decades, and treatment may cost up to USD 3.5 billion per year [6]. Among the bacteria that pose the greatest threat to world public health is methicillin-resistant *Staphylococcus aureus* (MRSA), where in particular, healthcare costs for a single specific serotype of *S. aureus*-caused infection reached almost EUR 9000 in Germany [7], and more than USD 18,000 in the US [8].

In general, *S. aureus* is one of the major opportunistic human pathogens [9], which has the ability to escape the immune system and can give rise to diversified infections ranging from superficial skin wounds to life-threatening sepsis [10]. Among the wide variety of infections, *S. aureus* is a well-known bacteria associated with wound infections, which generally colonize the outermost layer of wounds [11]. In particular, *S. aureus*-caused wound infections may be evaluated as a potential risk factor for MRSA concern [12], which has brought about the development of alternative antimicrobials substituted for traditional antibiotics. Moreover, *S. aureus* (especially MRSA) has the ability to adhere to living or inert surfaces, secreting an extracellular polymeric substance of proteins, polysaccharides, nucleic acids, and water, known as a biofilm. Subsequently, the biofilm matrix acts as a physical barrier that prevents the permeability of the drug into the bacterial community, and helps the microbe resist and minimize the effect of traditional antibiotics [13]. These challenges have given rise to a significant interest in the scientific community to develop herbal-based therapeutics with antimicrobial activity (e.g., essential oils) as a safer, green alternative to antibiotics [14].

Essential oils (EOs) are colored, aroma-rich, complex hydrophobic liquids [15], also known as volatile oils [16]. They are defined as the secondary metabolic product of aromatic plants [17] and are found in the various parts of plants such as flowers, roots, barks, stems, leaves, and seeds [18]. EOs are potent agents to diminish antimicrobial resistance [19] due to their significant therapeutic properties (i.e., antibacterial, antiseptic, and antioxidant activities) [20,21]. For this reason, EOs from pharmaceutical plants have also been examined as potent antimicrobial agents in animal production systems [22]. The antimicrobial activity of EOs does not only stem from their qualitative chemical composition, but also from the quantitative intensity of every single component that is included in the structure, as well as all plant-based products [23]. Their complex structure is mainly composes of terpenes (generally monoterpenes and sesquiterpenes) and terpenoids [24]. Even though some of these chemicals are water soluble, most of them are hydrophobic, so EOs are defined as hydrophobic [25,26].

Hydrophobicity is one of the most important features of an EO [16], enabling them to penetrate through the phospholipid-bilayer bacterial cell membrane after attaching to the cell surface [27]. As a consequence of the accumulation of EOs, the structure of the cell membrane may be destroyed, which results in an unfavorable change in the cell metabolism and causes the death of the cell [28]. It is also worth mentioning that the mechanism of action of EOs on the inhibition of bacterial growth is attributed to a series of reactions detrimental to bacterial cells that are defined as EO versatility [29]. EOs also exert anti-biofilm activity owing to both hydrophobic and hydrophilic moieties in their composition [30]. Accordingly, the hydrophobic components of EOs permeate the lipid substances of the cell membrane to diminish biofilm formation, while the hydrophilic ones diffuse through the exopolysaccharide matrix of the biofilm [31].

In this study, EOs of *Thymus sibthorpii*, *Origanum vulgare*, *Salvia fruticosa*, and *Crithmum maritimum* plants were chosen as the potential antimicrobial agents against various *S. aureus* strains to combat the antimicrobial resistance problem. All these species have already been used for traditional medications. Essential oils extracted from *Thymus* species are extensively used for pharmaceutical and cosmetic purposes with their various biological activities (e.g., antimicrobial and antioxidant activities) [32]. *Origanum vulgare* has been evaluated in preclinical studies for a long time thanks to its anti-inflammatory, antimicrobial, antioxidant, and anti-cancer properties [33]. EO of the *Salvia fruticosa* plant, which is one of the thousand species of the *Salvia* genus, is a traditional remedy for intestinal problems, epidermal problems, and gingivitis since ancient times [34,35]. *Crithmum maritimum* has not only been preferred for culinary purposes but has also been used for pharmaceutical and cosmetic reasons [36].

Thus, the chemical composition and the antimicrobial and anti-biofilm activity of *Thymus sibthorpii*, *Origanum vulgare*, *Salvia fruticosa*, and *Crithmum maritimum* EOs, extracted from freshly collected plants, were examined to identify potential antimicrobial and anti-biofilm agents. All EOs were tested against wild-type methicillin-sensitive and methicillin-resistant *S. aureus*, as well *S. aureus* ATCC 29213, bacteria, which have different antimicrobial resistance profiles. We hypothesized that if methicillin-sensitive and -resistant *S. aureus* strains do not differ in EO susceptibility, the selected EOs can be evaluated as alternative and safe players to combat the antimicrobial resistance problem. We strongly believe that with the present study, we filled this gap and make an important proposal since *S. aureus* is a reference species in the frontline of the resistance to antibiotics inquiry.

## 2. Results

The chemical composition of EOs was examined by GC-MS on a capillary column, and results are listed in Table 1 by their percentage of total presence. Twenty-eight, twenty-seven, thirty, and twenty-four compounds were identified in the *Thymus sibthorpii*, *Origanum vulgare*, *Salvia fruticosa*, and *Crithmum maritimum* EOs, respectively. The main chemical classes for EOs were monoterpene hydrocarbons, oxygenated monoterpenes, sesquiterpenes, and small amounts of alcohol, acetone, and quinone.

Carvacrol was detected as the major compound in *Thymus sibthorpii* and *Origanum vulgare* EOs with 52.62 and 78.72% of presence, while 1,8-cineol (39.70%) and β-phellandrene (28.01%) were the major substances in *Salvia fruticosa* and *Crithmum maritimum* EOs, respectively. Furthermore, the specific density of *Thymus sibthorpii*, *Origanum vulgare*, *Salvia fruticosa*, and *Crithmum maritimum* EOs was measured as 0.931, 0.932, 0.913, and 0.903 g/mL, respectively.

**Figure 1:**
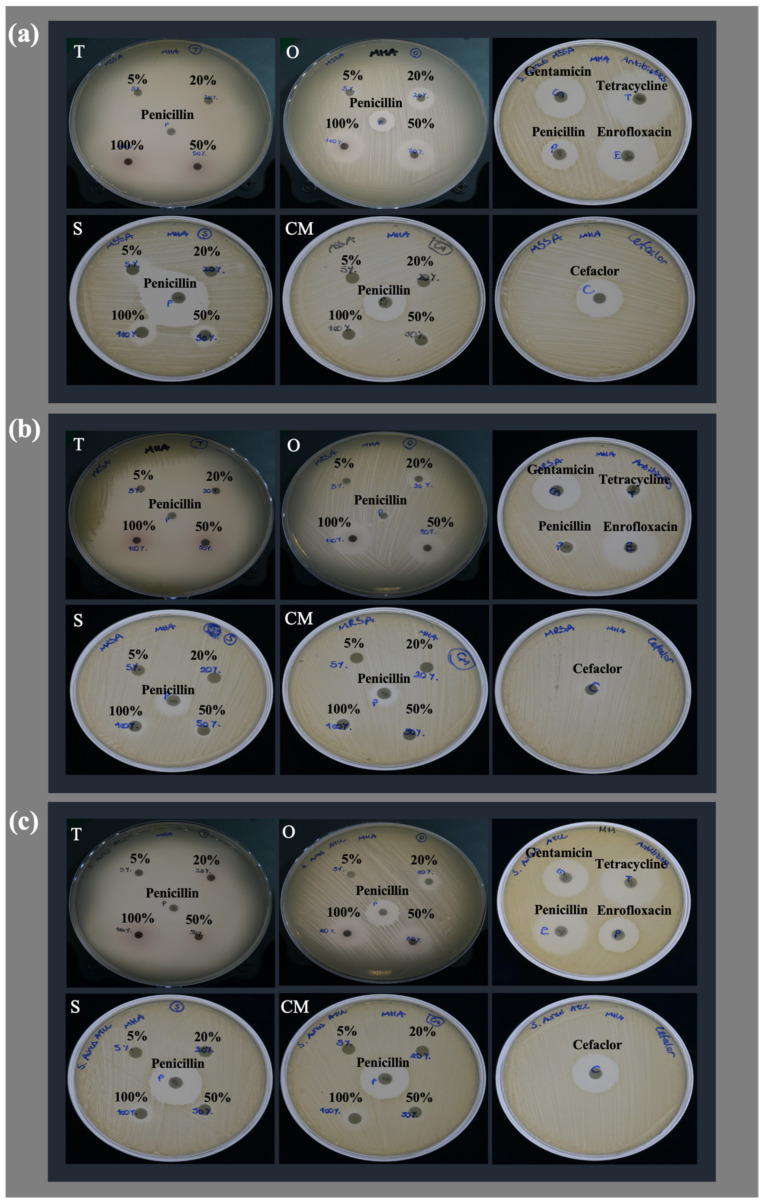
Qualitative illustration of inhibition zone diameters arising from testing EOs with different concentrations and reference antibiotics against (**a**) MSSA, (**b**) MRSA, and (**c**) *S. aureus* ATCC 29213.

Following the disc diffusion test, the inhibition zone diameters of varying concentrations of EOs and reference antibiotics are presented in Table 2. Among all tested antimicrobials, *Thymus sibthorpii* was found to be the strongest EO on all strains. Figure 1 shows the inhibition zone of each antimicrobial on each strain qualitatively. It can easily be seen that *Thymus sibthorpii* caused full inhibition on Mueller–Hinton agar plates for all microbial strains.

Table 2 demonstrates the MIC of the EOs and reference antimicrobials used on *S. aureus* strains. *Thymus sibthorpii* showed the lowest MIC for MSSA, whereas it has the same MIC as *Origanum vulgare* on MRSA and *S. aureus* ATCC 29213 strains. Contrary to this, the remaining EOs could not show lower MIC against all strains.

**Figure 2:**
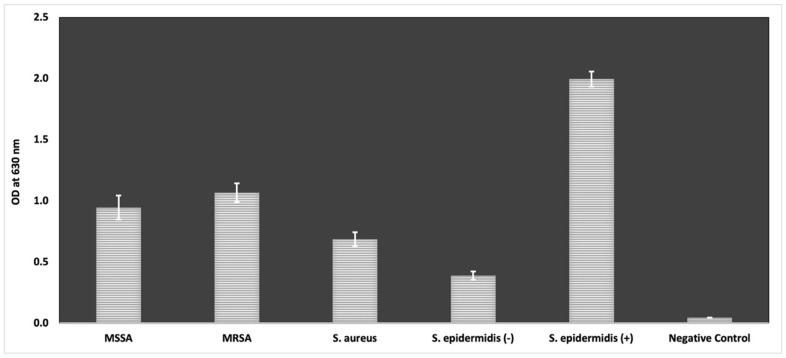
Comparison of biofilm formation ability of MSSA, MRSA, *S. aureus* ATCC 29213 (S. aureus), *S. epidermidis* ATCC 12228 (S. epidermidis (-)), and *S. epidermidis* ATCC 35984 (S. epidermidis (+)) regarding their OD values with negative control (TSBG medium only). Each value represents the mean of triplicate experiments with standard deviations (Tukey, *p* ≤ 0.05).

In the scope of the assessment of the inhibitory effect of antimicrobials on the biofilm formed by *S. aureus* cells, the biofilm formation capacity of these strains was examined. Figure 2 illustrates a comparison of the optical density of strains at 630 nm by modified microtiter plate biofilm formation assay. According to the results, while *S. epidermidis* ATCC 35984 showed the highest biofilm formation as expected, all tested *S. aureus* strains significantly produced biofilm.

All testing EOs inhibited the biofilm formed by *S. aureus* cells by about 95%, even at their half MIC. Gentamicin sulfate, which is the commonly used antimicrobial in the formulation of commercial antimicrobial and/or wound dressing products, could not show sufficient anti-biofilm activity at its MIC on testing strains (Table 3).

## 3. Discussion

There has been a remarkable interest in EOs as alternative antimicrobial agents to overcome microbial resistance issues in both humans and animals [37,38,39,40], which directly threaten public health [41]. Thus, the scientific community has shown substantial interest in antimicrobial activity screening methods [42]. The antimicrobial activity of EOs is examined by a variety of bioassays, such as Kirby–Bauer disc diffusion, agar well diffusion, bioautographic, agar dilution, and broth macro- and micro-dilution methods.

In the present study, *Thymus sibthorpii*, *Origanum vulgare*, *Salvia fruticosa*, and *Crithmum maritimum* EOs were assessed for their in vitro antimicrobial and anti-biofilm efficacy, as well as their chemical compositions. Their activities were also compared with the commonly used antimicrobials gentamicin sulfate [43], tetracycline hydrochloride [44], cefaclor [45], penicillin [46], and enrofloxacin [47]. Regarding the potency, according to the GC-MS results, the main bioactive component found is carvacrol for *Thymus sibthorpii* and *Origanum vulgare*. Eucalyptol and β-phellandrene were observed as the main compounds of *Salvia fruticosa* and *Crithmum maritimum* EOs, respectively.

Among the antimicrobial susceptibility tests, disc diffusion is a widely used method for the antimicrobial screening of plant-derived materials (e.g., EOs) [48], with its cost efficiency and convenience for evaluating a wide range of antimicrobials and microbes. In our study, *Thymus sibthorpii* was proven as the most effective EO against all tested methicillin-sensitive and -resistant *S. aureus* strains, followed by *Origanum vulgare*, which showed higher bacterial growth inhibition, against the same strains, than *Salvia fruticosa* and *Crithmum maritimum*, even at their lower concentrations (Table 2, Figure 2). Remarkably, 20% (*v*/*v*) of *Thymus sibthorpii* exhibited higher inhibition than all tested concentrations of other EOs and reference antibiotics on three of the tested *S. aureus* strains. However, *Salvia fruticosa* and *Crithmum maritimum* did not demonstrate a significant effect on the inhibition of *S. aureus* strains, a finding similar to previous findings in the literature [34,49]. This effect might be due to there being less of the active components present in these plants compared to *Thymus sibthorpii* and *Origanum vulgare* EOs. Houta et al. (2015) likewise reported that *Crithmum maritimum* EOs that were extracted from different plant parts did not present sufficient antimicrobial activity [49]. However, there were some differences between our results and some other studies. In one study, the antimicrobial activity of *Origanum vulgare* EO was screened against different *S. aureus* isolates by evaluating inhibition zone diameters and MIC values [50]. While zone diameters were generally higher than our results, the MIC of this EO as revealed in our work is significantly lower than the reported MIC values. This can be explained by differences in the composition of EOs even from the same plant species due to several factors affecting the chemical composition of EOs, such as harvesting season, climate, type of soil, and plant age [51]. Therefore, the antimicrobial activity of the same EOs may vary in different studies. To the best of our knowledge, the antimicrobial activity of *Thymus sibthorpii* EO has not been reported in the literature. However, the antimicrobial activity of crude extracts of *Thymus sibthorpii* was studied against the *S. aureus* bacterium, and the inhibition zone diameters were reported to be in the range of 9–15 mm [52], which was found to be higher for the EO in the present study.

According to the breakpoints of antibiotics reported by CLSI, penicillin, tetracycline [53], and cefaclor [54], MRSA appears resistant to them. For instance, if the zone diameter of tetracycline is equal to or larger than 19 mm on *Staphylococcus* species, then this microorganism can be evaluated as sensitive since its inhibition zone was evaluated as 10.426 mm in our work. Moreover, all tested *S. aureus* strains presented resistance against penicillin. Even though resistance was observed for most antibiotics, both methicillin-sensitive and -resistant *S. aureus* strains did not differ in their susceptibility to *Thymus sibthorpii*, which may indicate its power to combat microbial resistance.

Despite its several advantages, disc diffusion is not a suitable method to examine the MIC of antimicrobials since it is a qualitative assay and does not allow for evaluating the amount of penetrated antimicrobials into the agar media. Thus, the broth microdilution method was performed to assess the MIC of each EO and the reference antibiotics. The lowest concentration of an antimicrobial that can fully inhibit the growth of a microbial in microwells/tubes is defined as the MIC [55]. The MIC of *Thymus sibthorpii* for each strain was found to be 0.091 mg/mL, which is the same as the MIC of *Origanum vulgare* for MRSA and *S. aureus* ATCC 29213, whereas its MIC is 0.182 mg/mL for MSSA. This indicates the antimicrobial strength of these two EOs in lower concentrations. Surprisingly, while *Origanum vulgare* did not inhibit bacterial growth as effectively as *Thymus sibthorpii* in the disc diffusion method, they both showed similar MICs according to the broth microdilution method, which may be related to the qualitative nature of the disc diffusion method.

*Salvia fruticosa* exhibited 2.853 mg/mL MIC for all strains, which is two-fold lower than the MIC value of *Crithmum maritimum* for each strain. In other words, it can be said that a higher concentration of *Crithmum maritimum* is needed to complete bacterial growth inhibition towards *Salvia fruticosa*. In contrast to our findings, Kulaksiz and their team revealed that pure *Origanum vulgare* and *Salvia fruticosa* EOs presented more than 50% (*v*/*v*) MIC values against *S. aureus* ATCC 25923 [56].

The resistance of MRSA to the reference antibiotics may be observed by comparing their MIC with the methicillin-sensitive strain, as in the disc diffusion method. According to CLSI breakpoints, tetracycline and cefaclor were determined to be resistant and intermediate antimicrobials, respectively [53,54]. Even though these antibiotics did not demonstrate susceptibility for MRSA, as opposed to MSSA and *S. aureus* ATCC 29213 strains, which is consistent with CLSI documents, all the EOs exhibited the same level of activity for all strains. This outcome may also reveal the potency of EOs as a possible solution to microbial resistance.

It is believed that the substantial antimicrobial activity of the *Thymus sibthorpii* and *Origanum vulgare* EOs results from their main active ingredients, carvacrol and p-cymene, as well the contribution and synergism of other constituents. The major compounds of these EOs are carvacrol and p-cymene in different percentages of their content (Table 1). The phenolic monoterpenoid carvacrol is one of the most studied active compounds for antimicrobial activity [23]. It leads to an increase in bacterial cell membrane permeability and fluidity by damaging the cell membrane both functionally and structurally [57,58]. Moreover, it was reported that carvacrol may give rise to changes in the fatty acid composition [59] and transportation of cytoplasmic membrane ions, releasing of lipopolysaccharides [60,61], and alteration on cell membrane proteins and periplasmic enzymes [62,63]. On the other hand, the carvacrol precursor p-cymene was observed to increase the antimicrobial activity of single compounds present in EOs, such as carvacrol [23,64]. Although p-cymene cannot alter the membrane permeability and fluidity, it may cause a reduction in the melting point and enthalpy of the cell membrane [65], which can increase the impurity of the membrane.

**Figure 3:**
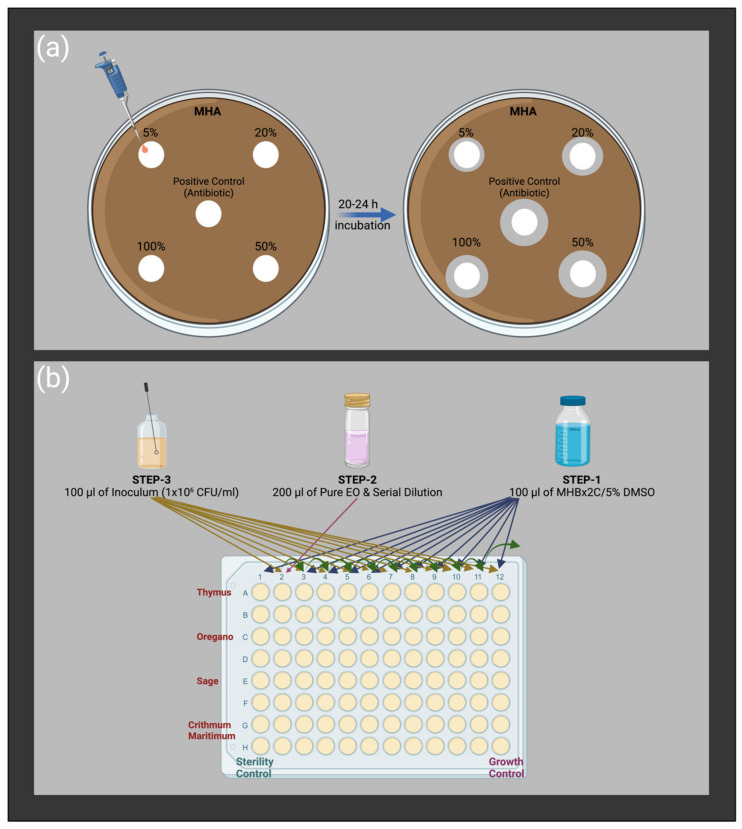
Schematic illustration of the experimental procedure of (**a**) disc diffusion and (**b**) broth microdilution methods.

*S. aureus* strains tested in our work exhibited strong biofilm formation (Figure 3). All tested EOs indicated a remarkable level of biofilm inhibition at their half MIC values against all three strains. Moreover, for *Salvia fruticosa* and *Crithmum maritimum,* which did not show higher growth inhibition like *Thymus sibthorpii*, their half MIC provided a sufficient level of biofilm inhibition. It was stated that both hydrophobic and hydrophilic components of EO are effective to exert anti-biofilm activity, while hydrophobic constituents are the main ones for inhibiting the growth of bacterial cells [30,66]. Therefore, the higher effectivity of testing EOs on the inhibition of biofilm formation compared to their antimicrobial activity might be explained by this phenomenon. In another respect, gentamicin was not found as an antimicrobial agent to inhibit the formation of biofilm by *S. aureus* cells, perhaps due to the antimicrobial resistance of the testing strains to gentamicin. For instance, gentamicin presented about 95% anti-biofilm activity on MRSA, which is a higher concentration, equivalent to a concentration of 1 mg/l. As a consequence, all EOs exerted good anti-biofilm activity on all *S. aureus*-formed biofilms at relatively low concentrations.

## 4. Materials and Methods

### 4.1. Plant Material and Extraction of Essential Oils

Aerial parts from *Thymus sibthorpii*, *Origanum vulgare* sbsp. hirtum, *Salvia fruticosa*, and *Crithmum maritimum* were collected during the flowering season in 2021 from the experimental farm of the Laboratory for Protection and Evaluation of Native species of the Institute of Plant Breeding and Genetic Resources (IPB&GR), preserved in Thessaloniki, Greece. The biomass was dried under ambient temperature in shade and subjected to distillation for 1.5 h for *Origanum vulgare* sbsp. hirtum and 1 h for the three other species, using a 50 L pilot-scale steam distillatory unit under steam pressure of 1.2 atm. The essential oils were collected and separated in a Florentine flask, dried over anhydrous sodium sulfate, and stored at 4–6 °C until further analysis [67]. Living mother plants and herbarium specimens of the species used for the production of EOs for experimentation are maintained at the collection of the Balkan Botanic Garden of Kroussia, Institute of Plant Breeding and Genetic Resources, Hellenic Agricultural Organization (ELGO)—DIMITRA, with the following unique IPEN (International Plant Exchange Network) accession numbers: *Thymus sibthorpii* GR-1-BBGK-01,1796, *Origanum vulgare* subsp. hirtum GR-1-BBGK-03,2107, *Salvia fruticosa* GR-1-BBGK-04,2411, and *Crithmum maritimum* GR-1-BBGK-97,719. The specific density of each fresh EO was measured by using a 10 mL pycnometer at 25 °C [68].

### 4.2. Identification of the Chemical Composition of Essential Oils

The essential oils were analyzed by gas chromatography–mass spectroscopy (GC-MS) on a capillary HP-5MS column (Agilent, Santa Clara, CA, USA), using a gas chromatograph 17A Ver. 3 interfaced with a mass spectrometer Shimadzu QP-5050A supported by the GC/MS Solution Ver. 1.21 software, using the method described previously [69]. The conditions of analysis were as follows: injection temperature, 260 °C; interface heating, 300 °C; ion source heating, 200 °C; EI mode, 70 eV; scan range, 41–450 amu; and scan time, 0.50 s. Oven temperature programs: (a) 55–120 °C (3 °C/min), 120–200 °C (4 °C/min), 200–220 °C (6 °C/min), and 220 °C for 5 min; and (b) 60–240 °C at 3 °C/min; carrier gas He, 54.8 kPa, split ratio 1:30. The relative content of each compound was calculated as percent of the total chromatographic area. The identification of the compounds was based on a comparison of their retention indices (RI) relative to n-alkanes (C7-C22) with corresponding literature data, and by matching their spectra with those of MS libraries (NIST 98, Wiley, Hoboken, NJ, USA) [70].

### 4.3. Antimicrobial Susceptibility Test and Bacterial Strains

The antimicrobial activity of *Thymus sibthorpii*, Origanum vulgare, *Salvia fruticosa*, and *Crithmum maritimum* EOs were screened against MSSA, MRSA, and *S. aureus* ATCC 29213 by the Kirby–Bauer disc diffusion and broth microdilution methods. The modified microtiter plate biofilm assay was also performed to assess the biofilm formation ability of tested strains, and the anti-biofilm activity of EOs, as well as reference antimicrobials. *Staphylococcus epidermidis* (*S. epidermidis*) ATCC 12228 and *S. epidermidis* 35984 were used as negative and positive quality control strains, respectively, for this bioassay. The wild-type MSSA and MRSA were previously derived from goat milk in our laboratory [71], and the other strains were purchased from American Type Culture Collection (ATCC).

#### 4.3.1. Antimicrobial Activity

##### Disc Diffusion Method

The CLSI M02-A11 document [72] was followed for the disc diffusion test, as schematically described in Figure 1a. Penicillin, enrofloxacin, gentamicin sulfate, tetracycline hydrochloride, and cefaclor (Oxoid, Hampshire, UK) were examined as reference antimicrobials. Briefly, the bacterial cells were grown in blood agar media overnight at 37 °C. Then, the inoculum was prepared in a sterile saline solution (bioMérieux, Marcy-l’Étoile, France) by adjusting the McFarland unit to 0.5 (~1 × 10^8^ CFU/mL) with fresh colonies. Afterward, the prepared inoculum was immediately spread out on dried Mueller–Hinton agar (Oxoid, Hampshire, UK) plates. The 6 mm diameter sterile Whatman paper N.1 discs were placed with 5, 20, 50, and 100% (*v*/*v*) of each EO diluted in 5% (*v*/*v*) dimethyl sulfoxide, DMSO (Honeywell, Charlotte, NC, USA), as well commercial antibiotic discs. EOs on paper discs were air-dried for half an hour, and plates were incubated at 37 °C overnight. At the end of the incubation period, images of each plate were taken, and inhibition zone diameters were evaluated using ImageJ software (version 2.0.0) by measuring the zone diameter of each disc a minimum of ten times from different points. Each condition was tested with three independent experiments.

##### The Modified Broth Microdilution Method

The broth microdilution method was studied according to the CLSI M07-Ed11 document with slight modifications [73] to assess the minimum inhibitory concentration (MIC) of each EO and the reference antimicrobials (gentamicin sulfate, tetracycline hydrochloride, and cefaclor). We used 5% (*v*/*v*) DMSO diluted in double-strength Mueller–Hinton broth (Fluka-Honeywell, Charlotte, NC, USA) as growth media for cells. Firstly, cells were grown on blood agar (Fluka-Honeywell, US) media and adjusted to a final concentration of 5 × 10^5^ CFU/mL utilizing sterile saline solution to prepare the inoculum. In a related row of 96-well plates, the first and last wells were defined as sterility and growth control, respectively. Serial dilution was performed by transferring 100 µL of well-mixed EO suspension to the other, and 100 µL of freshly prepared inoculum was added to the wells, except for the sterility control group. The concentration range was between 100% and 0.0488% (*v*/*v*) for EOs and between 128 and 0.000488 µg/mL for reference antibiotics. The 96-well plates were incubated in a horizontally shaking incubator at 37 °C and 75 rpm for 20 h, then re-incubated for 2 h after 1% (*w*/*v*) triphenyl tetrazolium chloride, TTC (Merk, Rahway, New Jersey, US), Gram stain transferring to each well. The red color indicated the living cells in the relevant well, and MIC was recorded as the concentration of the well just before the first red-colored well. Each test was repeated by three independent experiments. The experimental procedure is schematically described in Figure 1b.

#### 4.3.2. Anti-Biofilm Activity

##### Modified Microtiter Plate Biofilm Formation Assay

The biofilm formation ability of three *S. aureus* strains and the anti-biofilm activity of EOs and reference antimicrobials were assessed by microtiter plate biofilm formation assay [74,75] with some modifications. A flat-bottom 96-well microtiter plate (Sarstedt, Nümbrecht, Germany) was utilized for the analysis.

We mixed 100 µL of tryptic soy broth (Millipore Sigma, Burlington, UK) supplemented with 1% (*w*/*v*) glucose (TSBG) with 100 µL of inoculum, which was adjusted to a final concentration of 5 × 10^5^ CFU/mL, with fresh colonies grown on blood agar overnight at 37 °C by utilizing sterile saline solution. We used 100 µL of adjusted concentration of EO instead of TSBG to screen the anti-biofilm activity of antimicrobials. Then, plates were incubated at 37 °C for 20–24 h without agitation, which allows the cells to adhere to the surface of the well, followed by dumping out the cells by turning the plate over. Afterward, wells were washed with 250 µL of sterile water twice to remove planktonic bacteria, and the attached cells were fixed with 200 µL of pure methanol (Honeywell, Charlotte, NC, USA) for 15 min. Next, fixed cells were stained with 200 µL of 0.4% (*w*/*v*) gentian violet, also called crystal violet (Sigma-Aldrich, Dorset, UK) for 5 min, and the excess stain was rinsed off by placing the plates under gently running tap water. Stained cells in air-dried plates were resolubilized by 160 µL of 33% (*v*/*v*) glacial acetic acid (Honeywell, Charlotte, NC, USA). Each well was mixed thoroughly to ensure resolubilization of the attached cells; then, 100 µL of suspension was transferred to a new sterile plate, and the optical density (OD) was read at 630 nm. The biofilm inhibition percentage of each antimicrobial was evaluated as shown in Equation (1). Three independent experiments were performed for each treatment.

Biofilm Inhibition% = [(OD_Positive Control_ − OD_Experimental_)/(OD_Positive Control_)] × 100 (1)

### 4.4. Statistical Analysis

The antimicrobial analyses were carried out with three independent experiments for each treatment. The data were presented as the mean ± standard deviation and subjected to the one-way analysis of variance (ANOVA) followed by Tukey’s HSD (honestly significant difference) test at *p* < 0.05. All statistical analyses were performed in SPSS Statistics 20 (IBM SPSS Statistics, Version 20.0. Armonk, NY, USA, IBM Corp).

## 5. Conclusions

Essential oils are prominent antimicrobial and anti-biofilm agents due to the presence of various active components in their composition. Alongside their antimicrobial activity, they have great potency to overcome microbial resistance. In the present study, *Thymus sibthorpii* and *Origanum vulgare* EOs demonstrated great activity in the inhibition of the growth of different *S. aureus* strains, as well as in the inhibition of biofilm formation of these strains. We believed that the strength of these two EOs stems from the high amount of carvacrol and p-cymene in their structure. Even though *Salvia fruticosa* and *Crithmum maritimum* did not show sufficient antimicrobial activity, they could inhibit the biofilm formation by almost 95% with their half MIC values. From another perspective, the tested EOs show great anti-biofilm activity, while gentamicin sulfate could not inhibit biofilm even at its double MIC. This study clearly elucidates the in vitro effectiveness of different EOs on different *S. aureus* strains and reveals the adaptation of safer alternatives to overcome the incremental microbial resistance problem.

## Figures and Tables

**Table 1:** The essential oil composition of *Thymus sibthorpii*, *Origanum vulgare*, *Salvia fruticosa*, and *Crithmum maritimum* isolated during the flowering period, including the percentage of components and the experimental (RI) and literature-based (RIL) retention indices.

*Thymus sibthorpii*				*Origanum vulgare*				*Salvia fruticosa*				*Crithmum maritimum*			
Compound	RI	RIL	%	Compound	RI	RIL	%	Compound	RI	RIL	%	Compound	RI	RIL	%
Carvacrol	1309	1298	52.62	Carvacrol	1309	1298	78.72	1,8-cineol	1036	1033	39.70	β-phellandrene	1034	1031	28.01
*p*-cymene	1029	1026	18.75	*p*-cymene	1029	1026	8.19	Camphor	1150	1143	12.39	Sabinene	975	976	20.96
Thymoquinone	1247	1249	6.71	γ-terpinene	1061	1062	2.11	β-thujone	1116	1114	7.54	γ-terpinene	1061	1062	18.69
β-caryophyllene	1413	1418	3.70	Myrcene	991	991	1.64	α-pinene	936	939	7.03	1,8-cineol	1036	1033	9.53
Thymol	1295	1290	2.15	β-caryophyllene	1413	1418	1.27	α-terpinyl acetate	1345	1346	6.72	Thymol methyl ether	1236	1235	4.07
Carvacrol methyl ether	1242	1244	1.98	α-terpinene	1020	1018	1.01	*p*-cymene	1029	1026	4.31	cis-β-ocimene	1040	1040	3.68
cis-sabinene hydrate	1062	1065	1.85	α-pinene	936	939	0.98	Camphene	953	953	4.11	*p*-cymene	1029	1026	3.55
β-bisabolene	1507	1509	1.74	cis-sabinene hydrate	1062	1065	0.62	3-octanone	988	986	3.26	Terpinen-4-ol	1183	1177	2.66
Thymohydroquinone	1558	1553	1.36	Terpinen-4-ol	1183	1177	0.55	β-pinene	980	980	2.35	α-pinene	936	939	2.42
Caryophyllene oxide	1593	1581	1.03	α-thujene	929	931	0.48	Limonene	1032	1031	2.27	α-terpinene	1020	1018	1.64
α-thujene	929	931	0.86	Borneol	1175	1165	0.42	α-terpineol	1187	1189	2.00	Myrcene	991	991	1.44
α-terpinene	1020	1018	0.74	1-octen-3-ol	985	978	0.38	α-thujone	1105	1102	1.27	α-terpinolene	1086	1088	0.91
1,8-cineol	1036	1033	0.57	α-humulene	1452	1452	0.30	Borneol	1175	1165	0.80	α-thujene	929	931	0.48
α-humulene	1452	1452	0.42	Thymol	1295	1290	0.28	β-caryophyllene	1420	1418	0.74	α-phellandrene	1008	1005	0.44
α-pinene	936	939	0.36	Limonene	1032	1031	0.27	Terpinen-4-ol	1183	1177	0.64	trans-β-ocimene	1050	1050	0.24
trans-sabinene hydrate	1103	1098	0.32	Camphene	953	953	0.25	Linalyl acetate	1257	1257	0.52	Allo-ocimene	1132	1129	0.23
Terpinen-4-ol	1183	1177	0.29	Caryophyllene oxide	1593	1581	0.24	δ-terpineol	1161	1162	0.47	β-pinene	980	980	0.20
Limonene	1032	1031	0.27	β-phellandrene	1034	1031	0.23	trans-pinocamphone	1159	1160	0.32	Bicyclogermacrene	1492	1494	0.14
1-octen-3-ol	985	978	0.22	α-phellandrene	1008	1005	0.18	Linalool	1104	1098	0.31	cis-2-*p*-menthen-1-ol	1120	1117	0.11
β-pinene	980	980	0.17	β-pinene	980	980	0.16	Caryophyllene oxide	1593	1581	0.18	α-terpineol	1187	1189	0.08
β-phellandrene	1034	1031	0.16	α-terpinolene	1086	1088	0.15	Viridiflorol	1590	1590	0.18	β-caryophyllene	1420	1418	0.08
trans-β-farnesene	1456	1458	0.12	δ-cadinene	1517	1524	0.13	Tricyclene	925	926	0.13	Camphene	953	953	0.07
Germacrene D	1478	1480	0.11	δ-3-carene	1010	1011	0.10	α-thujene	929	931	0.13	cis-sabinene hydrate	1062	1065	0.07
δ-cadinene	1517	1524	0.11	trans-β-farnesene	1456	1458	0.10	Aromadendrene	1434	1419	0.11	Caryophyllene oxide	1593	1581	0.02
Borneol	1175	1165	0.07	β-bisabolene	1507	1509	0.10	Viridiflorene	1491	1493	0.08				
Camphene	953	953	0.06	Germacrene D	1478	1480	0.08	cis-sabinene hydrate	1062	1065	0.07				
δ-3-carene	1010	1011	0.05	1,8-cineol	1036	1033	0.07	α-terpinene	1020	1018	0.06				
Spathulenol	1580	1576	0.05					1-octen-3-ol	985	978	0.05				
								γ-terpinene	1061	1062	0.05				
								β-bisabolene	1507	1509	0.05				

**Table 2:** Inhibition zone diameter and minimum inhibition concentration of essential oils and reference antibiotics on treating microorganisms. A 6 mm inhibition zone diameter indicates no activity, and ND means not determined. Each value represents the mean of triplicate experiments with standard deviations. Different superscripts (a–m) in the row differ significantly for each strain (Tukey, *p* ≤ 0.05).

Treatment	Disk Content	Methicillin-Sensitive *S. aureus*		Methicillin-Resistant *S. aureus*		*S. aureus* ATCC 29213	
		Zone Diameter (mm)	MIC (mg/mL)	Zone Diameter (mm)	MIC (mg/mL)	Zone Diameter (mm)	MIC (mg/mL)
*Thymus sibthorpii*	5%	13.968 ± 0.679 ^c,d^	0.091	15.527 ± 0.698 ^b^	0.091	32.415 ± 1.992 ^g^	0.091
	20%	61.645 ± 1.923 ^k^		68.970 ± 4.667 ^d^		60.908 ± 0.298 ^h^	
	50%	70.765 ± 6.283 ^l^		68.983 ± 2.340 ^d^		61.380 ± 0.490 ^h^	
	100%	78.913 ± 2.897 ^m^		70.128 ± 5.797 ^d^		69.353 ± 2.581 ^i^	
*Origanum vulgare*	5%	7.039 ± 0.388 ^a,b^	0.182	6.310 ± 0.046 ^a^	0.091	6.000 ± 0.000 ^a^	0.091
	20%	17.811 ± 0.342 ^d,e^		14.005 ± 0.260 ^b^		11.137 ± 0.093 ^c^	
	50%	24.960 ± 0.149 ^f,g^		22.778 ± 0.293 ^c^		17.122 ± 0.171 ^d^	
	100%	25.089 ± 0.253 ^f,g^		23.569 ± 0.318 ^c^		17.552 ± 0.080 ^d^	
*Salvia* *fruticosa*	5%	6.000 ± 0.000 ^a^	2.853	6.000 ± 0.000 ^a^	2.853	6.000 ± 0.000 ^a^	2.853
	20%	13.643 ± 0.494 ^c,d^		6.000 ± 0.000 ^a^		6.000 ± 0.000 ^a^	
	50%	14.289 ± 0.534 ^c,d^		8.213 ± 0.249 ^a^		7.149 ± 0.103 ^a,b^	
	100%	17.464 ± 0.253 ^d,e^		11.184 ± 0.209 ^a,b^		9.399 ± 0.148 ^b,c^	
*Crithmum maritimum*	5%	6.000 ± 0.000 ^a^	5.644	6.000 ± 0.000 ^a^	5.644	6.000 ± 0.000 ^a^	5.644
	20%	6.000 ± 0.000 ^a^		6.000 ± 0.000 ^a^		6.000 ± 0.000 ^a^	
	50%	9.407 ± 0.138 ^a,b,c^		6.471 ± 0.066 ^a^		7.011 ± 0.164 ^a^	
	100%	11.128 ± 0.201 ^b,c^		7.689 ± 0.236 ^a^		7.527 ± 0.133 ^a,b^	
Gentamicin	10 µg	30.348 ± 0.149 ^h^	0.00025	22.914 ± 0.134 ^c^	0.0005	20.948 ± 0.022 ^e^	0.00025
Tetracycline	30 µg	41.125 ± 0.220 ^j^	0.002	10.426 ± 0.187 ^a,b^	0.032	26.897 ± 0.188 ^f^	0.001
Cefaclor	30 µg	28.120 ± 0.052 ^g,h^	0.002	6.693 ± 0.097 ^a^	0.016	20.826 ± 0.048 ^e^	0.002
Penicillin	10 units	22.355 ± 0.129 ^e^	ND	8.476 ± 0.038 ^a^	ND	18.719 ± 0.113 ^d,e^	ND
Enrofloxacin	5 µg	36.118 ± 0.091 ^i^	ND	25.059 ± 0.091 ^c^	ND	24.690 ± 0.132 ^f^	ND

**Table 3:** Biofilm formation inhibition percentages of different concentrations of essential oils and different concentrations of reference antibiotics on treating microorganisms. Each value represents the mean of triplicate experiments with standard deviations. Different superscripts (a–c) in the row differ significantly for each strain (Tukey, *p* ≤ 0.05).

Treatment	Concentration	Methicillin-Sensitive *S. aureus*	Methicillin-Resistant *S. aureus*	*S. aureus* ATCC 29213
*Thymus sibthorpii*	x4 MIC	95.134 ± 0.053 ^c^	95.817 ± 0.097 ^c^	93.528 ± 0.073 ^b^
	x2 MIC	95.293 ± 0.053 ^c^	95.817 ± 0.097 ^c^	93.577 ± 0.042 ^b^
	MIC	95.275 ± 0.061 ^c^	95.786 ± 0.081 ^c^	93.359 ± 0.042 ^b^
	x1/2 MIC	95.364 ± 0.081 ^c^	95.364 ± 0.609 ^c^	93.577 ± 0.042 ^b^
*Origanum vulgare*	x4 MIC	94.306 ± 0.239 ^c^	95.786 ± 0.047 ^c^	93.528 ± 0.126 ^b^
	x2 MIC	95.170 ± 0.214 ^c^	95.770 ± 0.054 ^c^	93.455 ± 0.073 ^b^
	MIC	95.205 ± 0.110 ^c^	95.708 ± 0.027 ^c^	93.334 ± 0.183 ^b^
	x1/2 MIC	94.993 ± 0.152 ^c^	93.772 ± 1.001 ^c^	93.068 ± 0.373 ^b^
*Salvia fruticosa*	x2 MIC	94.253 ± 0.583 ^c^	95.380 ± 0.311 ^c^	93.140 ± 0.414 ^b^
	MIC	94.905 ± 0.186 ^c^	95.427 ± 0.027 ^c^	93.189 ± 0.168 ^b^
	x1/2 MIC	85.985 ± 12.555 ^c^	95.068 ± 0.241 ^c^	93.262 ± 0.374 ^b^
*Crithmum maritimum*	x2 MIC	95.081 ± 0.242 ^c^	95.583 ± 0.241 ^c^	93.043 ± 0.484 ^b^
	MIC	94.658 ± 0.692 ^c^	95.551 ± 0.124 ^c^	93.031 ± 0.364 ^b^
	x1/2 MIC	83.799 ± 7.710 ^c^	95.349 ± 0.216 ^c^	91.521 ± 1.505 ^b^
Gentamicin	x4 MIC	95.275 ± 0.162 ^c^	95.458 ± 0.540 ^c^	93.261 ± 0.183 ^b^
	x2 MIC	58.342 ± 13.212 ^b^	81.598 ± 1.935 ^b^	48.201 ± 16.185 ^a^
	MIC	32.198 ± 20.528 ^a^	77.899 ± 2.234 ^b^	57.994 ± 10.493 ^a^
	x1/2 MIC	43.957 ± 20.026 ^a,b^	69.860 ± 7.767 ^a^	58.745 ± 16.368 ^a^
Tetracycline	x4 MIC	95.240 ± 0.242 ^c^	95.833 ± 0.000 ^c^	93.552 ± 0.042 ^b^
	x2 MIC	95.187 ± 0.092 ^c^	95.754 ± 0.118 ^c^	93.504 ± 0.042 ^b^
	MIC	95.169 ± 0.061 ^c^	95.848 ± 0.071 ^c^	93.528 ± 0.192 ^b^
	x1/2 MIC	95.223 ± 0.170 ^c^	95.520 ± 0.450 ^c^	93.553 ± 0.151 ^b^
Cefaclor	x4 MIC	95.152 ± 0.061 ^c^	95.630 ± 0.275 ^c^	93.407 ± 0.210 ^b^
	x2 MIC	95.117 ± 0.200 ^c^	95.567 ± 0.282 ^c^	93.189 ± 0.294 ^b^
	MIC	95.205 ± 0.110 ^c^	95.770 ± 0.177 ^c^	92.462 ± 0.965 ^b^
	x1/2 MIC	92.508 ± 4.779 ^c^	95.817 ± 0.135 ^c^	83.106 ± 3.567 ^b^
Standard Error		1.973	0.738	1.483
ANOVA *p*-value		<0.001	<0.001	<0.001

## Data Availability

Data are contained within the article.

## References

[1] Harbarth S., Balkhy H.H., Goossens H., Jarlier V., Kluytmans J., Laxminarayan R., Saam M., Van Belkum A., Pittet D. (2015). Antimicrobial resistance: One world, one fight!. Antimicrob. Resist. Infect. Control..

[2] Rozos G., Skoufos I., Fotou K., Alexopoulos A., Tsinas A., Bezirtzoglou E., Tzora A., Voidarou C. (2022). Safety Issues Regarding the Detection of Antibiotics Residues, Microbial Indicators and Somatic Cell Counts in Ewes’ and Goats’ Milk Reared in Two Different Farming Systems. Appl. Sci..

[3] Christaki E., Marcou M., Tofarides A. (2020). Antimicrobial resistance in bacteria: Mechanisms, evolution, and persistence. J. Mol. Evol..

[4] Lee J.-H. (2019). Perspectives towards antibiotic resistance: From molecules to population. J. Microbiol..

[5] Liu W., Yang C., Gao R., Zhang C., Ou-Yang W., Feng Z., Zhang C., Pan X., Huang P., Kong D. (2021). Polymer Composite Sponges with Inherent Antibacterial, Hemostatic, Inflammation-Modulating and Proregenerative Performances for Methicillin-Resistant Staphylococcus aureus-Infected Wound Healing. Adv. Healthc. Mater.

[6] Hofer U. (2019). The cost of antimicrobial resistance. Nat. Rev. Microbiol..

[7] Hübner C., Hübner N.-O., Hopert K., Maletzki S., Flessa S. (2014). Analysis of MRSA-attributed costs of hospitalized patients in Germany. Eur. J. Clin. Microbiol..

[8] Filice G.A., Nyman J.A., Lexau C., Lees C.H., Bockstedt L.A., Como-Sabetti K., Lesher L.J., Lynfield R. (2010). Excess costs and utilization associated with methicillin resistance for patients with Staphylococcus aureus infection. Infect. Control Hosp. Epidemiol..

[9] Ke Y., Ye L., Zhu P., Zhu Z.J.F.i.M. (2022). The clinical characteristics and microbiological investigation of pediatric burn patients with wound infections in a tertiary hospital in Ningbo, China: A ten-year retrospective study. Front. Microbiol..

[10] Pollitt E.J., Szkuta P.T., Burns N., Foster S.J. (2018). Staphylococcus aureus infection dynamics. PLoS Pathog..

[11] Serra R., Grande R., Butrico L., Rossi A., Settimio U.F., Caroleo B., Amato B., Gallelli L., De Franciscis S. (2015). Chronic wound infections: The role of Pseudomonas aeruginosa and Staphylococcus aureus. Expert Rev. Anti-Infect. Ther..

[12] Almeida G.C.M., dos Santos M.M., Lima N.G.M., Cidral T.A., Melo M.C.N., Lima K.C. (2014). Prevalence and factors associated with wound colonization by Staphylococcus spp. and Staphylococcus aureus in hospitalized patients in inland northeastern Brazil: A cross-sectional study. BMC Infect..

[13] Idrees M., Sawant S., Karodia N., Rahman A., Health P. (2021). Staphylococcus aureus biofilm: Morphology, genetics, pathogenesis and treatment strategies. Int. J. Environ. Res..

[14] Taiwo M., Adebayo O. (2017). Plant essential oil: An alternative to emerging multidrug resistant pathogens. JMEN.

[15] Mishra A.P., Devkota H.P., Nigam M., Adetunji C.O., Srivastava N., Saklani S., Shukla I., Azmi L., Shariati M.A., Coutinho H.D.M. (2020). Combination of essential oils in dairy products: A review of their functions and potential benefits. LWT.

[16] Chouhan S., Sharma K., Guleria S. (2017). Antimicrobial activity of some essential oils—Present status and future perspectives. Medicines.

[17] Ni Z.-J., Wang X., Shen Y., Thakur K., Han J., Zhang J.-G., Hu F., Wei Z.-J. (2021). Recent updates on the chemistry, bioactivities, mode of action, and industrial applications of plant essential oils. Trends Food Sci. Technol..

[18] Maurya A., Prasad J., Das S., Dwivedy A.K. (2021). Essential oils and their application in food safety. Front. Sustain. Food Syst..

[19] Stefanakis M.K., Touloupakis E., Anastasopoulos E., Ghanotakis D., Katerinopoulos H.E., Makridis P. (2013). Antibacterial activity of essential oils from plants of the genus Origanum. Food Control.

[20] Cimino C., Maurel O.M., Musumeci T., Bonaccorso A., Drago F., Souto E.M.B., Pignatello R., Carbone C. (2021). Essential oils: Pharmaceutical applications and encapsulation strategies into lipid-based delivery systems. Pharmaceutics.

[21] Saad N.Y., Muller C.D., Lobstein A. (2013). Major bioactivities and mechanism of action of essential oils and their components. Flavour Fragr. J..

[22] Tzora A., Giannenas I., Karamoutsios A., Papaioannou N., Papanastasiou D., Bonos E., Skoufos S., Bartzanas T., Skoufos I. (2017). Effects of oregano, attapulgite, benzoic acid and their blend on chicken performance, intestinal microbiology and intestinal morphology. Poult. Sci. J..

[23] Nazzaro F., Fratianni F., De Martino L., Coppola R., De Feo V. (2013). Effect of essential oils on pathogenic bacteria. Pharmaceuticals.

[24] Masyita A., Sari R.M., Astuti A.D., Yasir B., Rumata N.R., Emran T.B., Nainu F., Simal-Gandara J. (2022). Terpenes and terpenoids as main bioactive compounds of essential oils, their roles in human health and potential application as natural food preservatives. Food Chem. X.

[25] Man A., Santacroce L., Iacob R., Mare A., Man L. (2019). Antimicrobial activity of six essential oils against a group of human pathogens: A comparative study. Pathogens.

[26] Martins M.A., Silva L.P., Ferreira O., Schröder B., Coutinho J.A., Pinho S.P. (2017). Terpenes solubility in water and their environmental distribution. J. Mol. Liq..

[27] Da Silva B.D., Bernardes P.C., Pinheiro P.F., Fantuzzi E., Roberto C.D. (2021). Chemical composition, extraction sources and action mechanisms of essential oils: Natural preservative and limitations of use in meat products. Meat Sci..

[28] Bhavaniramya S., Vishnupriya S., Al-Aboody M.S., Vijayakumar R., Baskaran D. (2019). Role of essential oils in food safety: Antimicrobial and antioxidant applications. GOST.

[29] Burt S. (2004). Essential oils: Their antibacterial properties and potential applications in foods—A review. Int. J. Food Microbiol..

[30] Rossi C., Chaves-López C., Serio A., Casaccia M., Maggio F., Paparella A. (2022). Effectiveness and mechanisms of essential oils for biofilm control on food-contact surfaces: An updated review. Crit. Rev. Food Sci. Nutr..

[31] Kostoglou D., Protopappas I., Giaouris E. (2020). Common plant-derived terpenoids present increased anti-biofilm potential against Staphylococcus bacteria compared to a quaternary ammonium biocide. Foods.

[32] Amiri H. (2012). Essential oils composition and antioxidant properties of three thymus species. eCAM.

[33] Pezzani R., Vitalini S., Iriti M. (2017). Bioactivities of *Origanum vulgare* L.: An update. Phytochem. Rev..

[34] Bahadirli N.P. (2022). Comparison of Chemical Composition and Antimicrobial Activity of Salvia fruticosa Mill. and S. aramiensis Rech. Fill. (Lamiaceae). J. Essent. Oil-Bear. Plants.

[35] Karousou R., Vokou D., Kokkini S. (1998). Variation of Salvia fruticosa essential oils on the island of Crete (Greece). Bot. Acta.

[36] Senatore F., Napolitano F., Ozcan M. (2000). Composition and antibacterial activity of the essential oil from Crithmum maritimum L.(Apiaceae) growing wild in Turkey. Flavour Fragr. J..

[37] Battisti M.A., Caon T., de Campos A.M. (2021). A short review on the antimicrobial micro-and nanoparticles loaded with Melaleuca alternifolia essential oil. J. Drug Deliv. Sci. Technol..

[38] Aljeldah M.M. (2022). Antioxidant and antimicrobial potencies of chemically-profiled essential oil from asteriscus graveolens against clinically-important pathogenic microbial strains. Molecules.

[39] Jaradat N. (2021). Phytochemical profile and in vitro antioxidant, antimicrobial, vital physiological enzymes inhibitory and cytotoxic effects of artemisia jordanica leaves essential oil from Palestine. Molecules.

[40] Chávez-González M., Rodríguez-Herrera R., Aguilar C., Rai K.K.M. (2016). Essential oils: A natural alternative to combat antibiotics resistance. Antibiotic Resistance. Mechanisms and New Antimicrobial Approaches.

[41] Martin I., Sawatzky P., Liu G., Mulvey M. (2015). STIs and sexual health awareness month: Antimicrobial resistance to Neisseria gonorrhoeae in Canada: 2009–2013. CCDR.

[42] Balouiri M., Sadiki M., Ibnsouda S.K. (2016). Methods for in vitro evaluating antimicrobial activity: A review. JPA.

[43] Zain K.J., Brad B.A., Al Kurdi S.B., Jumaa M.M.G., Alkhouli M. (2021). Evaluation of Antimicrobial Activity of β-tricalcium Phosphate/Calcium Sulfate Mixed-up with Gentamicin: In-Vitro Study. Int. J. Dent. Oral Sci..

[44] Kulshreshtha G., Critchley A., Rathgeber B., Stratton G., Banskota A.H., Hafting J., Prithiviraj B. (2020). Antimicrobial effects of selected, cultivated red seaweeds and their components in combination with tetracycline, against poultry pathogen Salmonella enteritidis. J. Mar. Sci. Eng..

[45] Tsekoura E., Helling A., Wall J., Bayon Y., Zeugolis D. (2017). Battling bacterial infection with hexamethylene diisocyanate cross-linked and Cefaclor-loaded collagen scaffolds. Biomed. Mater..

[46] Górniak I., Bartoszewski R., Króliczewski J. (2019). Comprehensive review of antimicrobial activities of plant flavonoids. Phytochem. Rev..

[47] Chokejaroenrat C., Sakulthaew C., Satchasataporn K., Snow D.D., Ali T.E., Assiri M.A., Watcharenwong A., Imman S., Suriyachai N., Kreetachat T. (2022). Enrofloxacin and Sulfamethoxazole Sorption on Carbonized Leonardite: Kinetics, Isotherms, Influential Effects, and Antibacterial Activity toward S. aureus ATCC 25923. Antibiotics.

[48] Das K., Tiwari R., Shrivastava D. (2010). Techniques for evaluation of medicinal plant products as antimicrobial agent: Current methods and future trends. J. Med. Plant Res..

[49] Houta O., Akrout A., Najja H., Neffati M., Amri H.J.J.o.E.O.B.P. (2015). Chemical composition, antioxidant and antimicrobial activities of essential oil from Crithmum maritimum cultivated in Tunisia. J. Essent. Oil Bear. Plants.

[50] De Lima Marques J., Volcão L.M., Funck G.D., Kroning I.S., da Silva W.P., Fiorentini Â.M., Ribeiro G.A. (2015). Antimicrobial activity of essential oils of Origanum vulgare L. and Origanum majorana L. against Staphylococcus aureus isolated from poultry meat. Ind. Crops Prod..

[51] Chan W.-K., Tan L.T.-H., Chan K.-G., Lee L.-H., Goh B.-H. (2016). Nerolidol: A sesquiterpene alcohol with multi-faceted pharmacological and biological activities. Molecules.

[52] Kunduhoglu B., Pilatin S., Caliskan F. (2011). Antimicrobial screening of some medicinal plants collected from Eskisehir, Turkey. Fresenius Environ. Bull..

[53] CLSI (2012). Performance Standards for Antimicrobial Susceptibility Testing: Twenty-fifth Informational Supplement—CLSI Document M100-S25.

[54] CLSI (2012). Performance Standards for Antimicrobial Susceptibility Testing: Twenty-second Informational Supplement—CLSI Document M100-S22.

[55] CLSI (2018). Methods for Dilution Antimicrobial Susceptibility Tests for Bacteria That Grow Aerobically: Approved Standard—Ninth Edition—CLSI Document M07-A9.

[56] Kulaksiz B., Sevda E., Üstündağ-Okur N., Saltan-İşcan G. (2018). Investigation of antimicrobial activities of some herbs containing essential oils and their mouthwash formulations. Turk. J. Pharm. Sci..

[57] Sikkema J., de Bont J.A., Poolman B. (1995). Mechanisms of membrane toxicity of hydrocarbons. Microbiol. Rev..

[58] Sharifi-Rad M., Varoni E.M., Iriti M., Martorell M., Setzer W.N., del Mar Contreras M., Salehi B., Soltani-Nejad A., Rajabi S., Tajbakhsh M. (2018). Carvacrol and human health: A comprehensive review. Phytother. Res..

[59] Di Pasqua R., Betts G., Hoskins N., Edwards M., Ercolini D., Mauriello G. (2007). Membrane toxicity of antimicrobial compounds from essential oils. J. Agric. Food Chem..

[60] Helander I.M., Alakomi H.-L., Latva-Kala K., Mattila-Sandholm T., Pol I., Smid E.J., Gorris L.G., von Wright A. (1998). Characterization of the action of selected essential oil components on Gram-negative bacteria. J. Agric. Food Chem..

[61] Kachur K., Suntres Z. (2020). The antibacterial properties of phenolic isomers, carvacrol and thymol. Crit. Rev. Food Sci. Nutr..

[62] Juven B., Kanner J., Schved F., Weisslowicz H. (1994). Factors that interact with the antibacterial action of thyme essential oil and its active constituents. J. Appl. Bacteriol..

[63] Churklam W., Chaturongakul S., Ngamwongsatit B., Aunpad R. (2020). The mechanisms of action of carvacrol and its synergism with nisin against Listeria monocytogenes on sliced bologna sausage. Food Control.

[64] Rattanachaikunsopon P., Phumkhachorn P. (2010). Assessment of factors influencing antimicrobial activity of carvacrol and cymene against Vibrio cholerae in food. JBB.

[65] Cristani M., D’Arrigo M., Mandalari G., Castelli F., Sarpietro M.G., Micieli D., Venuti V., Bisignano G., Saija A., Trombetta D. (2007). Interaction of four monoterpenes contained in essential oils with model membranes: Implications for their antibacterial activity. J. Agric. Food Chem..

[66] Shahabi N., Tajik H., Moradi M., Forough M., Ezati P. (2017). Physical, antimicrobial and antibiofilm properties of Zataria multiflora Boiss essential oil nanoemulsion. Int. J. Food Sci. Technol..

[67] Maloupa E., Krigas N. (2008). The Balkan Botanic Garden of Kroussia, Northern Greece. Sibbaldia Int. J. Bot. Gard. Hortic..

[68] Simirgiotis M.J., Burton D., Parra F., López J., Muñoz P., Escobar H., Parra C. (2020). Antioxidant and antibacterial capacities of Origanum vulgare L. essential oil from the arid Andean Region of Chile and its chemical characterization by GC-MS. Metabolites.

[69] Sarrou E., Tsivelika N., Chatzopoulou P., Tsakalidis G., Menexes G., Mavromatis A. (2017). Conventional breeding of Greek oregano (Origanum vulgare ssp. hirtum) and development of improved cultivars for yield potential and essential oil quality. Euphytica.

[70] Adams R.P. (2007). Identification of essential oil components by gas chromatography/mass spectrometry. Quadrupole Mass Spectroscopy.

[71] Fotou K., Tzora A., Voidarou C., Alexopoulos A., Plessas S., Avgeris I., Bezirtzoglou E., Akrida-Demertzi K., Demertzis P. (2011). Isolation of microbial pathogens of subclinical mastitis from raw sheep’s milk of Epirus (Greece) and their role in its hygiene. Anaerobe.

[72] CLSI (2015). Performance Standards for Antimicrobial Disk Susceptibility Tests: Approved Standard—CLSI Document M02-A11.

[73] CLSI (2006). Methods for Dilution Antimicrobial Susceptibility Tests for Bacteria That Grow Aerobically: Approved Standard—Eighth Edition—CLSI Document M07-A7.

[74] Stepanović S., Vuković D., Dakić I., Savić B., Švabić-Vlahović M. (2000). A modified microtiter-plate test for quantification of staphylococcal biofilm formation. J. Microbiol. Methods.

[75] Borges S., Silva J., Teixeira P. (2012). Survival and biofilm formation by Group B streptococci in simulated vaginal fluid at different pHs. ALJMAO.

